# IgGκ Signal Peptide Enhances the Efficacy of an Influenza Vector Vaccine against Respiratory Syncytial Virus Infection in Mice

**DOI:** 10.3390/ijms241411445

**Published:** 2023-07-14

**Authors:** Anastasia Pulkina, Kirill Vasilyev, Arman Muzhikyan, Mariia Sergeeva, Ekaterina Romanovskaya-Romanko, Anna-Polina Shurygina, Marina Shuklina, Andrey Vasin, Marina Stukova, Andrej Egorov

**Affiliations:** Smorodintsev Research Institute of Influenza of the Ministry of Health of the Russian Federation, 197022 St. Petersburg, Russia; pulkina.a@yandex.ru (A.P.);

**Keywords:** respiratory syncytial virus, vaccine design, influenza vector, regulatory T-cells

## Abstract

Intranasal vaccination using influenza vectors is a promising approach to developing vaccines against respiratory pathogens due to the activation of the mucosa-associated immune response. However, there is no clear evidence of a vector design that could be considered preferable. To find the optimal structure of an influenza vector with a modified NS genomic segment, we constructed four vector expressing identical transgene sequences inherited from the F protein of the respiratory syncytial virus (RSV). Two vectors were designed aiming at transgene accumulation in the cytosol. Another two were supplemented with an IgGκ signal peptide prior to the transgene for its extracellular delivery. Surprisingly, adding the IgGκ substantially enhanced the T-cell immune response to the CD8 epitope of the transgene. Moreover, this strategy allowed us to obtain a better protection of mice from the RSV challenge after a single intranasal immunization. Protection was achieved without antibodies, mediated by a balanced T-cell immune response including the formation of the RSV specific effector CD8+ IFNγ+/IL10+-producing cells and the accumulation of Treg cells preventing immunopathology in the lungs of infected mice. In addition to the presented method for optimizing the influenza vector, our results highlight the possibility of achieving protection against RSV through a respiratory-associated T-cell immune response alone.

## 1. Introduction

The respiratory syncytial virus (RSV) is one of the most clinically relevant respiratory pathogens, along with the influenza virus and SARS-CoV-2. Being a prevalent pathogen among children under five years old, RSV is a leading cause of infant hospitalization [1,2]. RSV infection also severely affects the elderly and high-risk adults. The disease burden of RSV infection is comparable to that of non-pandemic influenza A [3]. To the best of our knowledge, there is no licensed vaccine against RSV approved worldwide, only GSK’s Arexvy vaccine for older adults has recently been approved by the U.S. FDA [4].

Currently, the induction of a neutralizing antibody response is considered the primary goal of vaccination against most infectious diseases. However, RSV infection may still occur in the presence of a high titer of neutralizing antibodies [5], and a person can stay protected in the absence of detectable antibodies [6]. The induction of the T-cell response may be essential for protection against RSV [7], which can be confirmed by the data on a long and severe course of the infection in children with a defective T-cell response [7,8]. Nevertheless, systemic virus-specific T-cells may be insufficient for protection [9]. The formation of mucosa-associated IFNγ-producing CD8+ T-cells is necessary for protection against RSV [7,10,11]. However, RSV-specific IFNγ-producing CD8+ T-cells can also cause immunopathology, leading to disease exacerbation [12]. The formation of populations of specific CD8+ and CD4+ cells that simultaneously produce IFNγ and IL10 as well as the accumulation of Tregs is required to prevent cytotoxic T lymphocyte (CTL)-driven immunopathology in the lungs, as shown previously [13]. Thus, vaccines capable of inducing such cell subpopulations in the respiratory tract may prove to be the most effective.

It is well-established that the T-cell response in the respiratory tract can be induced by intranasal vaccine administration [10]. Among the platforms for mucosal immunization, influenza vectors delivering RSV antigens intranasally are of great interest because they induce protective immunity against both respiratory pathogens. The intranasal administration of attenuated influenza vectors is safe and immunogenic, particularly in terms of triggering the T-cell immune response against antigens linked to the non-structural protein 1 (NS1) [14,15,16]. These NS vectors show a prophylactic efficacy against bacterial infections such as tuberculosis [15,16,17] or brucellosis [18], and viral respiratory infections like RSV [19,20,21,22,23] or parainfluenza [24,25] in animal models. Immunization with influenza NS vectors contributes to the Th1 polarization of the immune response [16,26], which is beneficial for protection against RSV infection [27,28,29].

The protective efficacy of vector vaccines depends on the choice of a foreign antigen and its expression pattern. There are multiple options for constructing influenza NS vectors and it is unclear whether a protective immune response would benefit from either intracellular or extracellular expression of the RSV antigen. Transgenes linked to the influenza NS1 protein normally accumulate inside infected cells since NS1 has nuclear and cytoplasmic localization signals. In the present work, we constructed two types of influenza NS vectors and compared their immunogenicity and protective efficacy. The vectors expressed an identical cassette of two antigenic regions of the RSV F protein but differed in the mechanism of transgene processing. The first group of vectors (NS-F and NS-2AF) was designed for the intracellular accumulation of the transgene. The second group (NS-2AsF and sF-NS) promoted the transgene secretion, hence the F antigen was supplemented with the IgGκ signal peptide. We found that vectors carrying the IgGκ signal peptides provided better protection against RSV. Immunization with the IgGκ-containing vectors had an advantage in the induction of the RSV-specific CD8+ T-cell response and regulatory T-cell accumulation in the lungs after RSV infection.

## 2. Results

### 2.1. Construction of Influenza NS Vectors Encoding an RSV Antigen

**Figure 1:**
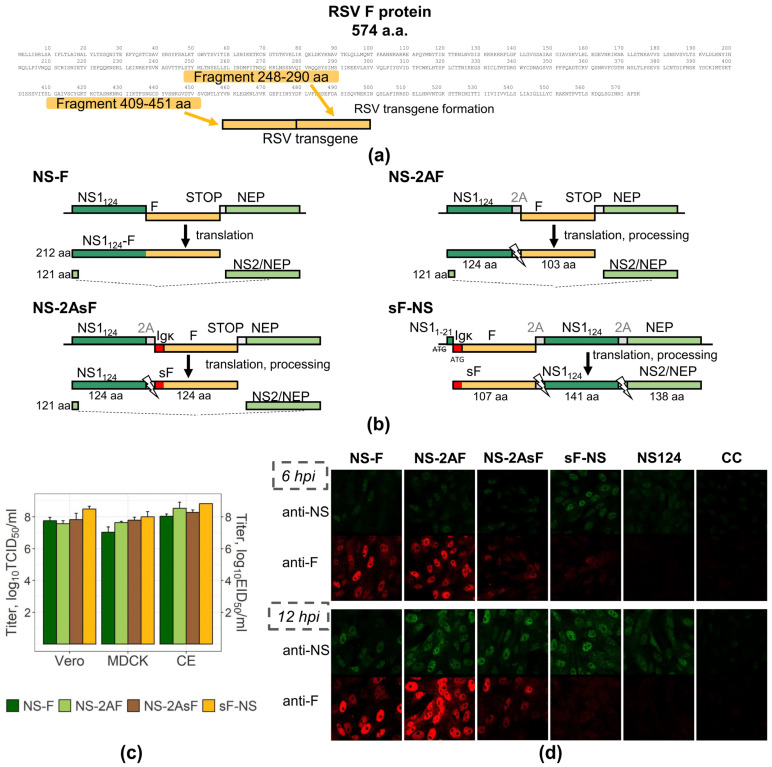
Construction and characterization of the chimeric NS gene segments. (**a**) Location of RSV F protein fragments selected for inclusion into the antigenic cassette. (**b**) Schematic map of the chimeric *NS* gene segments and expressed proteins. NS1_124_—non-structural protein of the influenza A/PR/8/34 virus truncated to 124 amino acids; F—transgene cassette of F_248–290_ and F_409–451_ RSV protein fragments; STOP—stop codon; NS2/NEP—nuclear export protein of the influenza A/PR/8/34 virus; PR8-NS11-21—nucleotide sequence encoding the first 21 amino acids of the NS1 protein modified by start codon removal; Igκ—sequence encoding the IgGκ signal peptide; 2A—sequence encoding the 2A-peptide; sF—secreted RSV transgene. (**c**) Reproductive properties of the influenza NS vectors. Virus replication in the Vero and MDCK cell lines and CEs (mean ± SD). (**d**) Expression of proteins by the influenza NS vectors in Vero cells upon infection. Immunofluorescent staining was performed 6 and 12 h after infection with the recombinant viral vectors or NS124 control virus and in the mock-infected cell control (CC). Polyclonal rabbit and mouse sera followed by secondary antibodies labelled with Alexa Fluor 488 and Alexa Fluor 568 dyes were used to visualize the NS1 protein (green) and the RSV transgene (red). The image was taken on a Leica TCS SP8 confocal microscope at ×60 magnification.

The fusion F glycoprotein of RSV is the target for neutralizing antibodies and CTLs. Therefore, to generate influenza vectors, we chose two highly conserved regions of the F protein: F_248–290_ and F_409–451_. The selected fragments are located in the antigenic sites II and IV [30] and contain the mouse F_249–258_ CD8 T-cell epitope. We optimized the nucleotide sequence of the F-antigen cassette (transgene) for the GC content corresponding to the human influenza virus. Figure 1a shows the structure of the transgene. The transgene sequence was used to construct four variants of the influenza A/Puerto-Rico/8/34-based (A/PR/8/34) vector attenuated by removing the C-terminal half of the NS1 protein (NS1_124_) [31].

The first group of vectors (NS-F and NS-2AF) was designed for the intracellular accumulation of the transgene. In the NS-F vector, the F antigen was linked directly to the N-terminal part (amino acids 1–124) of the truncated NS1_124_ protein (Figure 1b). Similar vectors were described and characterized previously as genetically stable and highly reproductive in various cell substrates [15,32,33]. The NS-2AF vector combined the N-terminus transgene sequence with the 2A “autocleavage” site for post-translational separation.

The second group of vectors (NS-2AsF and sF-NS) was constructed for the extracellular secretion of the transgene. In addition to the 2A site, the transgene in the NS-2AsF vector was supplemented with the sequence derived from the murine immunoglobulin kappa (IgGκ) light chain for extracellular delivery of the transgene. As is known, N-terminal signal peptides direct proteins to the endoplasmic reticulum membrane and initiate translocation into the lumen of the endoplasmic reticulum. The sF-NS vector was designed to further improve the transgene secretion efficacy. In this construct, the IgGκ signal peptide was introduced at the N-terminal part of the chimeric polyprotein, which included the F transgene followed by the NS1_124_ and NS2/NEP sequences separated by 2A sites. Here, the transgene and NS1_124_ were placed in reverse order compared to other constructs, and NEP expression was ensured by 2A cleavage without splicing.

The structure of the chimeric NS gene segments and proteins expressed is shown in Figure 1b. All viruses were successfully rescued in Vero cells using an 8-plasmid reverse genetic system [34]. All constructed vectors were genetically stable and carried the transgene of the correct size over seven consecutive passages in chicken embryos (CEs) or Vero cells, as confirmed by RT-PCR and sequencing. The vectors could be amplified to high titers (8.0–9.0 log_10_ EID_50_/mL) in 10-day-old embryonated chicken eggs and in cell lines suitable for vaccine manufacturing (7.5–8.0 log_10_ TCID_50_/mL) (Figure 1c).

### 2.2. Transgene Expression Patterns in Infected Vero Cells

To evaluate the expression of the vector transgene and its intracellular location in infected Vero cells, we applied immunofluorescent staining using NS1- or transgene-specific antiserum. The NS124 virus lacking the transgene was used as a control (Figure 1d).

Infection of Vero cells with the NS-F or NS-2AF vector promoted cytoplasmic accumulation of the transgene, which was detected as early as 6 h after the infection and was followed by an increase in signal intensity at the 12-h time point. In contrast, vectors containing the IgGκ signal peptide demonstrated a weak specific signal in 6 and 12 h, presumably due to the extracellular delivery of the transgene or its rapid degradation in the cytosol. The intensity of the NS1-specific signal was similar for both the modified and control viruses, indicating that synthesis and intracellular localization of NS1_124_ was not affected by the attachment of foreign sequences.

### 2.3. Immunization with the NS-2AsF and sF-NS Vectors Provides Enhanced T-Cellular Immune Response in Mouse Lungs

**Figure 2:**
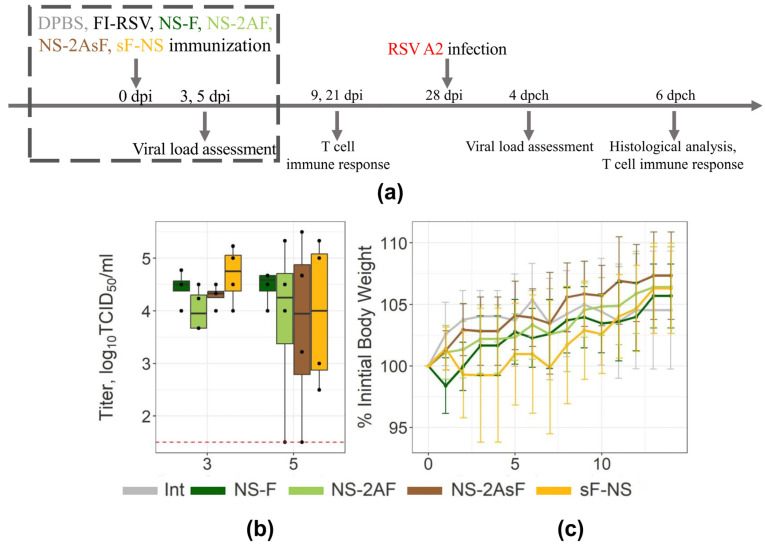
Attenuation of influenza NS vectors in mice. (**a**) Mice experiment scheme. (**b**) Boxplots represent the mean viral load in mouse lungs (*n* = 4). Mice were infected with 6.0 log10 EID50 of the vectors. Viral titer in lung suspension was determined on days 3 and 5. The red dotted line indicates the detection limit. Black dots indicate individual values. Data are representative of two experiments with four mice per group. (**c**) Weight dynamics of mice immunized with vectors at 6.0 log10 EID50 is presented as the mean values for each group (mean ± SD, *n* = 12). Data are representative of four experiments with 7–12 mice per group.

To evaluate the attenuation of influenza NS vectors, Balb/c mice were inoculated with the vectors at the dose of 6.0 log_10_ TID_50_/animal. The scheme of the experiment is shown in Figure 2a. The ability of the vectors to replicate in vivo was assessed in the lungs on days 3 and 5 after intranasal inoculation; the weight dynamics was monitored for 14 days. We observed similar viral loads in the lungs in all groups of immunized mice on the third and fifth days after immunization; however, there was a wide variation in values on the fifth day (Figure 2b). These data indicate that the constructed vectors had similar growth patterns in mouse lungs. The dynamics of weight loss was also similar in all experimental groups and did not exceed a 5% reduction from the baseline (Figure 2c). Thus, all vectors showed similar levels of attenuation in mice.

Immunization with all vectors induced similar seroconversion in response to the influenza virus (64–128 HAI titer) but did not elicit any detectable IgG antibodies to RSV in the serum blood sample, as evaluated using the sorbed F protein or purified RSV ELISA. Thus, supplementation of the transgene with the IgGκ signal peptide provoked no B-cell response to the transgene.

**Figure 3:**
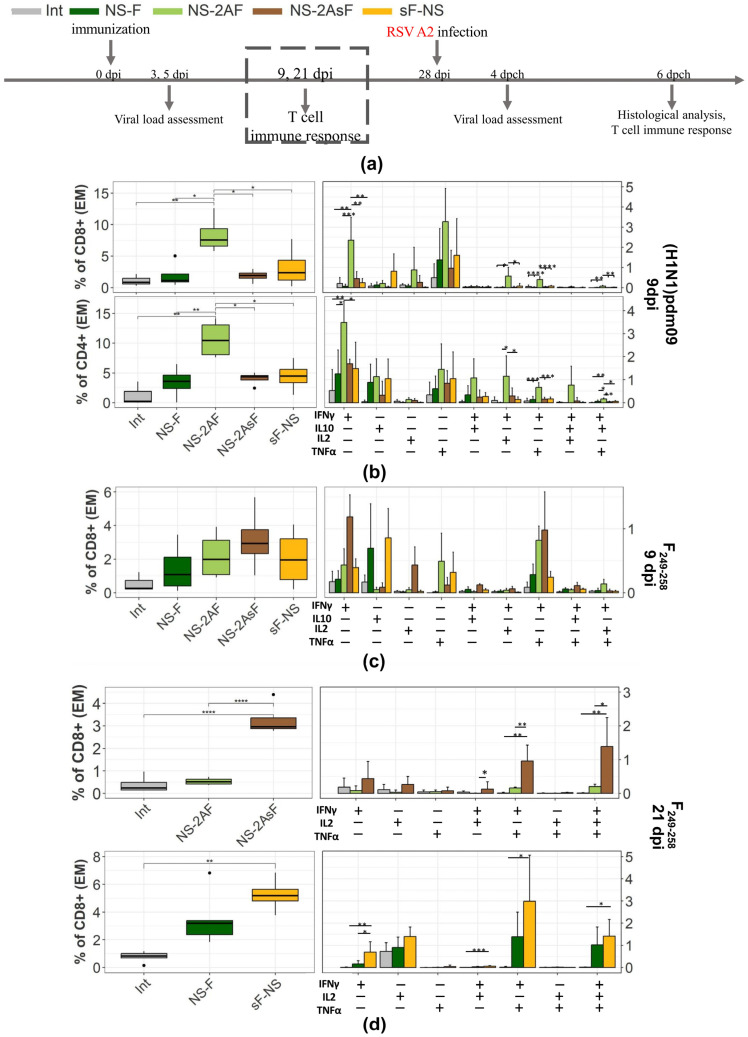
Adaptive T-cell immune response in mice after intranasal immunization with the influenza vectors. (**a**) Mice experiment scheme. (**b**) Boxplots represent the cumulative sum of all the effector memory CD8+ and CD4+ T lymphocytes producing at least one cytokine IFNγ/IL10/IL2/TNFα in mouse lungs 24 h after in vitro stimulation with the (H1N1)pdm09 influenza strain. Bar charts with error bars represent the percentage of different subpopulations of cytokine-producing T-cells. (**c**,**d**) Boxplots represent the cumulative sum of all the CD8+ Tem lymphocytes producing at least one cytokine in mouse lungs 9 (**c**) or 21 (**d**) days after intranasal immunization (dpi). Bar charts with error bars represent the percentage of different subpopulations of cytokine-producing T-cells. Restimulation with the RSV F_249–258_ epitope was performed for 6 h. Groups were compared using ANOVA followed by Tukey’s post hoc test (*: *p* < 0.05, **: *p* < 0.01, ***: *p* < 0.001, ****: *p* < 0.0001, *n* = 4, mean ± SD). Data are representative of two experiments with 4–5 mice per group.

To assess the development of the T-cell immune response, the lungs of immunized mice were collected on days 9 and 21 after immunization and used for the extraction of lymphocytes. The antigen-specific CD8+ effector memory T-cell (Tem) immune response was estimated by restimulation with the influenza (H1N1) pdm09 virus (Figure 3b) or the F_249–258_ RSV-epitope (Figure 3c,d). The gating strategy used for the analysis is presented in Figure S1. Typical plots of intracellular staining for the production of cytokines are shown in Figures S2 and S3.

The pattern of the T-cell immune response against influenza virus antigens was different. The NS-2AF vector without the IgGκ was found to be significantly more immunogenic toward influenza antigens compared to the other vectors. A statistically significant difference between the NS-2AF group and other experimental groups was found for both the СD4+ and СD8+ T-cells (Figure 3b).

All vectors stimulated the accumulation of CD8+ T lymphocytes specific to the RSV epitope in the lungs. Interestingly, the most pronounced RSV-specific response was found in the NS-2AsF group. Although the NS-2AsF vector was the most immunogenic for the RSV epitope, it induced the lowest numbers of influenza-specific CD8+ and CD4+ cells in the lungs (Figure 3b). The population composition of cytokine-producing CD8+ T lymphocytes formed in response to the RSV stimulation is shown in Figure 3c (ninth day) and Figure 3d (21st day). On day 9 after immunization, antigen-specific cells were mainly represented by monofunctional IFNγ+IL10-IL2–TNFα-, IFNγ-IL10+IL2–TNFα-, and IFNγ-IL10-IL2–TNFα+ T-cells and by polyfunctional IFNγ+IL10-IL2–TNFα+ CD8+ T-cells in all groups. A statistically significant difference was shown only for the polyfunctional IFNγ+IL10+IL2–TNFα-T cell subpopulation between the NS-2AsF and NS-2AF groups. The tendency toward an increased percentage of other subpopulations was mainly observed in the NS-2AsF group. This was especially pronounced for the subpopulation of monofunctional IFNγ+IL10-IL2-TNFα-T cells. On the 21st day after immunization, the difference demonstrating the advantage of IgGκ-containing vectors was even more pronounced (Figure 3d). We conducted two series of experiments in which we compared NS-2AF with NS-2AsF or NS-F with sF-NS. A statistically significant increase in polyfunctional IFNγ+IL2–TNFα+ and IFNγ+IL2+TNFα+ T cell subpopulations was found in both NS-2AsF and sF-NS. No significant differences were found in the number of RSV-specific CD4+ cells in the lungs (Figure S4).

Thus, inserting the signal peptide at the N-terminal end of the transgene, especially in the NS-2AsF design, enhanced the T-cell immunogenicity of the RSV-specific antigen but did not promote the immunogenicity of the influenza-specific antigens carried by the vector.

### 2.4. Immunization with the NS-2AsF and sF-NS Vectors Provides Better Protection against RSV Infection

To evaluate the protective efficacy of the constructed vectors, a challenge experiment was performed in Balb/c mice. FI-RSV was used as a vaccine preparation that is known for its ability to induce vaccine-associated enhanced respiratory disease [35,36]. The control mock-immunized group of mice received DPBS. Mice were challenged with the RSV A2 strain four weeks after a single intranasal immunization (intramuscular for FI-RSV). Viral load was assessed on day 4, histopathological changes were examined on day 6.

Body weight loss did not exceed 5% in any of the experimental groups for 6 days after infection.

**Figure 4:**
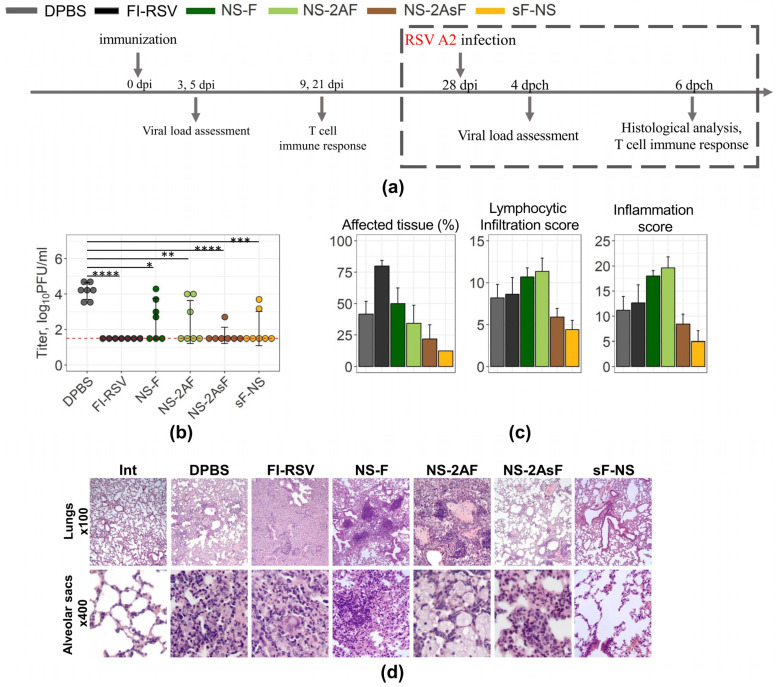
Protection in mice. (**a**) Mice experiment scheme. (**b**) Viral load in the lungs of the control and immunized mice 4 days after intranasal challenge with the RSV A2 virus. The results are presented as individual values for each animal with the mean ± SD for each group. Results were analyzed by ANOVA followed by Tukey’s post hoc test. Asterisks indicate a statistically significant difference between groups (* *p* < 0.05, ** *p* < 0.01, *** *p* < 0.001, **** *p* < 0.0001). The red dotted line marks the detection limit. Data are representative of four experiments with 4–7 mice per group (**c**) Bar charts with error bars from the examination of lung tissue from the immunized and control mice after RSV challenge. Percentages of affected tissue and scores of lymphocytic infiltration and inflammation were evaluated 6 days after infection (mean ± SD, *n* = 4). Data are representative of three experiments with four mice per group. (**d**) Microphotographs of hematoxylin and eosin-stained histological sections of lungs represent the most pronounced pathological changes in the vaccinated and control mice.

In the lungs of the mock-immunized mice, the viral load remained within the range of 3.5–4.5 log_10_ PFU/mL (Figure 4b). The lung injury in mock-immunized mice was typical for a murine model of RSV infection [37]. This was mainly characterized by moderate inflammatory infiltration of the alveolar septa with the involvement of granular leukocytes and the accumulation of alveolar macrophages and lymphocytes in the alveoli lumen (Figure 4c,d). Mononuclear cells moderately infiltrated the interstitial, peribronchial, and peribronchiolar tissue. Additionally, perivascular infiltrates and mild vasculitis were detected. Slight hypertrophy and hyperplasia of the epithelium of the air-bearing section of the lungs were accompanied by limited intraepithelial infiltration with the manifestation of bronchitis (Figure 4c,d).

As expected, immunization with the FI-RSV vaccine preparation prevented virus reproduction but triggered the most pronounced histological lesions [19,22,23,35]. The observed lung tissue damage pattern after FI-RSV vaccine application was reported previously [19,22,23,35] and differed from that of the mock-immunized group, predominantly by the involvement of eosinophils in the inflammatory process (Figure 4c,d).

The vectors differed in reducing both the viral load and the severity of the RSV-induced histopathology in the lungs. A statistically significant decrease in the viral load was observed in all groups of immunized mice. The NS-F vector had the least effect on RSV reproduction (decrease in the viral load reached 1.57 log_10_ PFU/mL (Figure 4b)) and did not prevent histopathology, except for a decrease in the number of granular leukocytes involved in the inflammatory process (Figure 4d).

Immunization with the NS-2AF vector reduced the viral load by 1.74 log_10_ PFU/mL (Figure 4b). The lung damage was characterized by epithelium damage with the accumulation of cellular debris in the lumen of bronchioles, the formation of large foci of collapsed lung tissue, and the accumulation of alveolar macrophages in the alveoli.

Immunization of mice with the IgGκ-containing NS-2AsF and sF-NS vectors resulted in a more pronounced reduction in the viral load (2.5 log_10_ PFU/mL and 2.09 log_10_ PFU/mL, respectively), as shown in Figure 4b. In the NS-2AsF-immunised mice, low inflammation scores and minimal lymphocytic infiltration were observed (Figure 4c). Negligible histological changes with no signs of inflammatory lesions were observed in mice from the sF-NS group (Figure 4c,d).

Thus, considering the decreased viral load and fewer pathological changes, immunization with the vectors containing the IgGκ signal peptide appears beneficial for protection against RSV infection.

**Figure 5:**
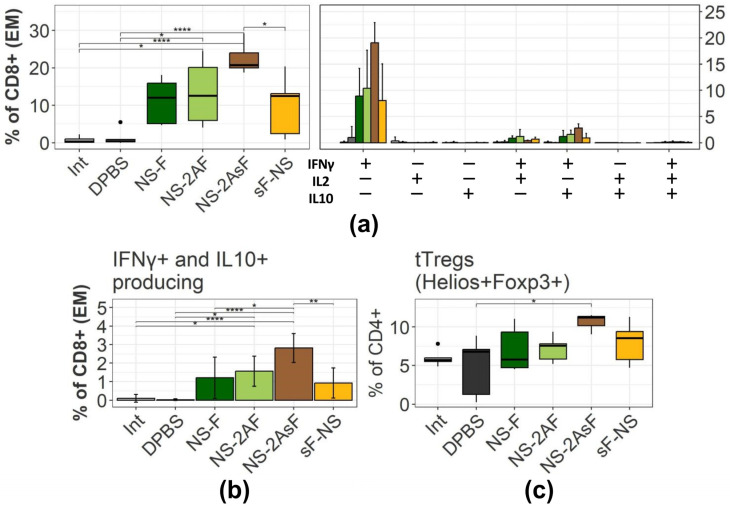
Adaptive T-cell immune response in mice lungs 6 days after RSV infection. (**a**) Boxplots on the left represent the cumulative sum of all the effector memory CD8+ T-cells producing at least one cytokine IFNγ/IL2/IL10 6 h after in vitro stimulation with the RSV F_249–258_ epitope. Bar charts with error bars represent the percentage of different subpopulations of cytokine-producing T-cells. (**b**) Histogram represents the percentage of IFNγ+/IL2-/IL10+ subpopulations of cytokine-producing T-cells within the CD8+ T-cell population. (**c**) Boxplots represent the percentage of regulatory T-cells in the lungs of immunized mice 6 days after the RSV challenge. Asterisks indicate a statistically significant difference between groups (mean ± SD, *: *p* < 0.05, **: *p* < 0.01, ****: *p* < 0.0001). Groups were compared using ANOVA followed by Tukey’s post hoc test (*n* = 5). Data are representative of two experiments with 4–5 mice per group.

To compare T-cell memory recall after RSV infection, we measured different T-cell populations in the lungs on day 6 after the challenge. Figure 5a illustrates the percentages of cytokine-producing T-cells after in vitro restimulation with the RSV F_249–258_ epitope. The gating strategy used for the analysis is presented in Figure S1. An example of typical plots of intracellular staining for the production of cytokines is shown in Figure S5a. All groups of mice immunized with influenza vectors demonstrated extensive CD8+ response against the RSV F_249–258_ epitope encoded by the transgene. There was no increase in the number of these cells in the DPBS control group. We observed a statistically significant increase in the T-cell response in the NS-2AsF group (Figure 5a), which coincided with the trend observed after immunization. Additionally, we evaluated the vectors’ ability to induce the formation of a population of effector cells capable of co-producing IFNγ and IL10. Figure 5b illustrates the advantage of the NS-2AsF vector in stimulating these cells.

The influence of the vaccination on the accumulation of Treg cells in the lungs upon RSV infection was also analyzed using intracellular staining for the FOXP3 and Helios transcription factors (Figure 5c, Figures S5 and S6). We demonstrated that immunization with the IgGκ-containing vectors led to a prominent accumulation of Helios+Foxp3+ Treg cells in mouse lungs. A statistically significant difference was shown between the NS-2AsF and the infection control groups.

We can conclude that a single intranasal immunization with all the constructed influenza vectors promoted an antigen-specific CD8+ T-cell immune response after the RSV challenge. However, we observed that the NS-2AsF construct, which demonstrated superior protectivity according to the virological and histological findings, also triggered a more pronounced formation of Treg cells and RSV-specific IFNγ+/IL10+ producing CD8+ cells after the RSV challenge.

## 3. Discussion

Influenza virus vectors are a promising approach to developing vaccines against respiratory infections. The intranasal route of vaccination makes it possible to induce both B- and T-cell-mediated mucosal immunity in the respiratory tract. Numerous articles describe various options for engineering influenza virus vectors carrying transgenes introduced into the HA [19,38], NA [17,20,21], PB2 [25], or NS genomic segments [15,16,21,22]. Among these approaches, influenza NS vectors that express protective antigens in the NS1 open reading frame are particularly interesting since the inserted transgenes do not affect the virion morphology. In addition, influenza NS vectors carrying an altered Interferon antagonist protein NS1 are known for their increased ability to stimulate the T-cell immune response due to their self-adjuvant effect upon intranasal administration [32,39]. However, the vectors with the NS1-linked transgenes cannot effectively deliver the antigen to the extracellular compartment, and thus these transgenes may exhibit limited B-cell immunogenicity. Rather than find an ideal protective antigen for the RSV vaccine, this study aimed primarily to optimize the design of influenza NS vectors and to increase the protective efficacy of the vectors in a murine model of RSV infection.

We constructed several influenza vectors expressing the same fragments of the RSV F protein conserved regions (248–290 and 409–451 amino acids). An influenza vector expressing a similar transgene F_243–294_ in the hemagglutinin and providing protection against RSV infection was previously described [19]. Here, we constructed influenza NS vector variants with and without the IgGκ signal peptide that carries the 2A autoproteolysis site and the F transgene located upstream or downstream of the truncated NS sequences. By applying these modification methods, we generated four genetically stable vectors that demonstrated similar levels of attenuation in mice. We then tested their prophylactic efficacy against RSV infection after a single intranasal immunization of Balb-c mice. We obtained conclusive evidence of a higher efficiency of vectors in which the insertion of the RSV antigen was supplemented with the signal peptide.

To understand the mechanism behind the variation in vector efficacy, we analyzed the parameters of the immune response after immunization and after the challenge. The first obvious finding was the absence of a detectable antibody response to the RSV antigen after immunization with either construct. This indicates that supplementation of the antigen with the signal peptide did not enhance the B-cell response. In contrast, a previous study demonstrated that adding a signal peptide to a Dengue virus vaccine candidate based on the recombinantly modified vaccinia virus Ankara contributed to the development of an antibody response to the target protein in immunized mice [40]. Perhaps, in our case, the antigen-specific antibodies were not formed due to the rapid degradation of the chimeric protein in the cytosol. It is also possible that the introduced transgene is too short to activate the B-cell response, or the B-cell response could not reach the systemic compartment, and therefore, the antibody response was not detected in the blood serum.

When examining the T-cell response in the lungs on day 9 after immunization, we found that the vectors induced comparable RSV-specific immune responses toward the F_249–258_ epitope. However, the tendency toward the highest percentage of CD8+ Tem cells in the lungs was identified for the NS-2AsF vector. The most pronounced differences between NS-2AsF and the other groups were found for the monofunctional IFNγ+IL10-IL2-TNFα- and polyfunctional IFNγ+IL10+IL2-TNFα-T cell subpopulations. At the same time, NS-2AsF was weaker than the NS-2AF vector, which lacks the signal peptide, in terms of inducing the T-cell response to influenza virus antigens. On day 21, the difference in the CD8+ cells’ specific response to the RSV F_249–258_ epitope became substantial, indicating the great immunogenicity advantage of both vectors carrying the signal peptide. Thus, attachment of the signal peptide to the transgene triggered refocusing of the CD8+ Tem response to the RSV epitope due to a better presentation of the antigen in the context of MHC I. It has previously been noted that signal peptide insertion can shift immunogenicity, causing a more pronounced response to the insert and making the immunorecessive insert more dominant [41]. Our results are consistent with those published earlier, where adding a signal peptide was shown to enhance the Т-cell immune response to an inserted foreign antigen [40,42,43]. Indeed, through immunofluorescent staining, we found that the F antigen quickly disappeared from the cytoplasm of the infected cells if the vector carried the IgGκ signal peptide. This observation could also indicate a combination of different processing mechanisms, leading to higher immunogenicity of the vectors modified by adding the IgGκ signal peptide.

The improved presentation of the RSV F_249–258_ epitope in the context of MHC I, which was mediated by the signal peptide, could be attributed to several reasons. First, when secreted successfully outside of an infected cell, the peptide may become available for dendritic cells that cross-present it to the CD8+ T lymphocytes. Some RSV epitopes including F_249–258_ used for T-cell stimulation in our work were only presented through MHC-I-mediated cross-presentation when the CTLs were primed, presumably by the exogenous proteasome-independent TAP-independent lysosomal pathway [44,45]. However, it should be noted that we could not detect the transgene in the supernatant of the infected cells. The addition of brefeldin A to the medium during infection of the cell culture also did not result in a higher transgene expression, which suggests that brefeldin A does not affect the processing of the chimeric protein. Second, it cannot be ruled out that the signal peptide, especially with a proline at the N-terminus exposed after 2A cleavage, may be suboptimal for effective protein delivery to the ER; incorrectly folded or misassembled proteins may promote its degradation on proteasomes followed by TAP-dependent or independent endogenous presentation [46]. It is known that artificial proteins that contain hydrophobic sequences can be unstable and undergo accelerated degradation in proteasomes, thus enhancing the peptide supply of MHC I [46,47,48]. Signal peptides exert several other effects on the distribution of proteins aside from their secretion outside the cell [49]. Therefore, we hypothesize that the improved immunogenicity of the NS-2AsF and sF-NS vectors may result from multiple mechanisms.

The results of the challenge experiments carried out after a single intranasal immunization with four vectors allowed us to draw several conclusions. First, none of the influenza constructs were able to cause immunopathology in the lungs that would be typical of the vaccine preparation FI-RSV. Second, the FI-RSV vaccine achieves its ability to suppress the reproduction of RSV at the cost of destroying lung tissue, making this an unreliable criterion for vaccine effectiveness. In contrast, the influenza vectors with almost similar antiviral efficacy ensured the absence of the vaccine-associated enhanced respiratory disease effect. Third, even small variations in the structure of a viral vector can make a significant difference in the effectiveness of the vaccine. A statistically significant decrease in viral load was found when comparing all immunized groups to the mock-immunized mice. However, only the mice immunized with the NS-2AsF or sF-NS vector demonstrated minimal histological changes in the lungs, while mice in the NS-F and NS-2AF groups showed pronounced lesions typical for a murine model of RSV infection.

Our results are consistent with those published previously, where immunization with influenza vectors was reported to reduce the viral load in the lungs following RSV infection [19,22]. The obtained data on the RSV virus isolation from the lungs of mice correlated with the data on the T-cell immune response to the RSV transgene. However, the protection mediated by vectors containing the IgGκ signal peptide may not only be associated with the amplitude of the T-cell response to the RSV antigen, but also with the quality of the effector cells.

It is well-known that an unbalanced T-cell immune response can be harmful due to an excessive reaction of CD8+ T-cells, which leads to lung tissue damage [12,50], or a Th2-biased CD4+ T-cell response resulting in excessive mucus production and airway hyperresponsiveness [27,28,29,50,51,52]. It is also well-established that the Th1-polarized immune response mediates viral infection control via IFNγ and TNFα cytokine production, ensuring efficient viral clearance [53,54,55]. The Th2-biased immune response, on the other hand, develops after immunization with a formalin-inactivated RSV vaccine, causing an exacerbation upon natural infection [36]. The disease severity not only depends on the Th1/Th2 disproportion, but also on other immune mechanisms such as achieving balance with the help of Treg cells [56]. The induction of Treg cells facilitates the suppression of an excessive immune response that contributes to tissue damage [52,57]. Thus, a successful vaccine against RSV should induce antigen-specific CD8+ CTLs in the respiratory tract, balanced by appropriate Treg activity.

It has also been shown that IL10, produced by effector T-cells, plays a critical role in the control of lung inflammation during RSV infection [58], and a proportion of these cells is able to co-produce IFNγ [13,58]. IFNγ has been demonstrated to suppress inflammation in previously vaccinated RSV-infected mice [59]. However, IFNγ in RSV-infected mice may be useful in limited amounts only, and its production and anti-inflammatory effects should be tightly regulated by IL10 expression. Therefore, it is necessary to achieve a delicate balance between the elimination of the virus and the control of the immune response in the respiratory tract.

We assessed the adaptive immune response after the challenge to understand immunological mechanisms promoting the increased protective efficacy of the IgGκ vectors. Again, no antibody immune response to the transgene was found in any group of immunized animals, indicating the importance of the protective T-cell mediated immunity. A more pronounced and robust RSV-specific Т-cell immune response was consistently found in mice immunized with the NS-2AsF vector. Consistent with this, immunization with the best-performing NS-2AsF vector was found to stimulate the formation of the highest percentage of CD8+ effector T-cells co-producing IFNγ and IL10.

It is worth noting that we found more Treg cells in the NS-2AsF-immunized mice upon RSV infection. It was previously shown that higher levels of Treg cells after vaccination might suppress the development of vaccine-associated enhanced respiratory disease in the lungs after RSV infection [60,61]. Our data indicate that the vector supplemented with the IgGκ signal peptide is more potent in triggering the accumulation of Treg cells in mouse lungs upon subsequent RSV infection. Given that antigen-dependent differentiation of Treg cells implies the MHC-II-mediated presentation of extracellular antigens, our results agree with the theoretical expectations of a better performance of the IgGκ-containing vectors in inducing the Treg formation. However, the question of the antigen-specific nature of the Treg accumulation deserves further investigation.

## 4. Materials and Methods

### 4.1. Cells

Vero cells (ATCC #CCL-81, Manassas, VA, USA) adapted to growth in a serum-free medium were cultured in OptiPro medium (Gibco, Waltham, MA, USA) supplemented with 2% GlutaMax (Gibco, USA). Hep-2 cells (ATCC #CCL-23, Manassas, VA, USA) were maintained in DMEM (Biolot, St. Petersburg, Russia) supplemented with 5% SC-Biol fetal bovine serum (Biolot, Russia). MDCK cell culture (IRR, #FR-58, USA) was cultivated in AlphaMEM (Biolot, Russia) supplemented with 5–10% Sc-Biol.

### 4.2. Recombinant Influenza Viruses

A previously described method of reverse genetics was used to produce recombinant influenza vectors expressing the RSV antigen [34]. Four bidirectional plasmids encoding a chimeric NS1 protein were synthesized de novo and cloned into the pHW2006 vector (Evrogen, Moscow, Russia). The sequences of the obtained plasmid constructs were confirmed by sequencing. The chimeric plasmids and the plasmids encoding the remaining seven genes of the A/Puerto Ricco/8/1934 (A/PR/8/34) virus were used to transfect Vero cells using the Nucleofection II system (Lonza #VCA-1003, Basel, Switzerland ). Viruses were propagated in 10–12-day-old chicken embryos (CEs) (JSC “Poultry Farm Sinyavinskaya”, Priladozhsky Village, Russia). RT-PCR on specific primers for the *NS* gene UTR regions and gene sequencing were performed to control the genetic stability of viral strains and the presence of the heterologous insert.

The control virus PR8-NS124 (NS124), carrying the NS1 protein truncated to 124 amino acids, was obtained from the collection of the Smorodintsev Research Institute of Influenza.

The infectious activity of the viruses was assessed by titration in CEs, Vero cells, or MDCK cells, as described elsewhere. The Reed–Muench method was used to calculate the 50% infectious dose [62].

### 4.3. Respiratory Syncytial Virus

The RSV serotype A strain A2 (RSV A2) was obtained from the International Reagent Resource (IRR, #FR-294). The virus was cultured in HEp-2 cells in DMEM supplemented with 2% Sc-Biol and 1% antibiotic-antimycotic (Gibco, USA).

The infectious activity of RSV was determined by a plaque assay in 24-well culture plates (Nunc, Roskilde, Denmark) with an 80%-monolayer of Hep-2 cells. A series of tenfold dilutions was prepared in a viral growth medium and added in a volume of 250 µL per well. After an hour of incubation, the semi-liquid coating with 0.3% agarose (NBCo, Thorold, ON, Canada,) in MEM medium (Gibco, USA), supplemented with 2% serum, 1% antibiotic-antimycotic, and 1% GlutaMAX, was added to the plates. The cells were fixed with 80% acetone in PBS four days after incubation. Immunostaining was performed with the monoclonal mouse 4F2 antibodies specific to RSV [63] and the goat anti-mouse IgGs (H+L) labeled with horseradish peroxidase (Abcam, Cambridge, UK). For the plaque visualization, a solution of 0.05% DAB (Serva, Heidelberg, Germany) and 0.015% hydrogen peroxide in PBS was used. The RSV viral titer was expressed in decimal logarithms of plaque-forming units as log_10_ PFU/mL.

Formalin-inactivated RSV (FI-RSV) was obtained following the protocol described earlier [35]. Briefly, the RSV A2 virus was accumulated in HEp-2 cell culture for 72 h. After freezing, the cell suspension was centrifuged at 5000× *g* and +4 °C for 40 min. The supernatant was filtered through a 450 nm membrane. A total of 1:4000 formalin (0.009%) was added before the second incubation for 72 h at 37 °C. The virus was purified through a 30% sucrose cushion by ultracentrifugation in an SW-41 rotor at 36,000 rpm (~190,000× *g*, +4 °C, 4 h). The pellet was resuspended in a serum-free medium. Protein concentration was measured using the Quant-iT Protein Assay Kit (Thermo, Waltham, MA, USA) with a Qubit fluorometer (Invitrogen, Waltham, MA, USA). To produce a prototype of the FI-RSV vaccine, aluminum hydroxide (Sigma-Aldrich, Burlington, MA, USA) was added 1 h before immunization.

### 4.4. Immunofluorescent Staining

Vero cells were grown on coverslips until they reached a monolayer, then infected with the recombinant viruses at the dose of 1 TCID_50_/cell and incubated for 6–12 h. Cells were fixed with 4% paraformaldehyde (Sigma Aldrich, USA) and permeabilized with 0.2% Triton X-100 (Helicon, Moscow, Russia). Immunostaining was performed using a polyclonal mouse serum to the F antigen, a rabbit serum to the NS1 protein, and the corresponding fluorescently labelled secondary antibodies—Goat Anti-Mouse IgG (Alexa Fluor^®^ 568) and Goat Anti-Rabbit IgG H&L (Alexa Fluor^®^ 488). Animal serum was obtained after intraperitoneal immunization with the NSl124 protein or the F peptide. The samples were photographed with a Leica TCS SP8 confocal microscope (Leica Microsystems, Wetzlar, Germany). The photos were processed using the Leica Application Suite X software (LAS X Office 1.4.4).

### 4.5. Laboratory Animals

Balb/c mice (female, 6–8-week-old) were obtained from the Nursery for Laboratory Animals Pushchino (Shemyakin and Ovchinnikov Institute of Bioorganic Chemistry RAS, Moscow, Russia). All animal studies followed the international recommendations (Directive 2010/63/EU) and the protocols approved by the Bioethics Committee of the Smorodintsev Research Institute of Influenza.

### 4.6. Immunization and RSV Challenge in Mice

To compare vector reproduction and strain attenuation, Balb/c mice were immunized intranasally with 6.0 log_10_ TCID_50_ of the recombinant virus vectors in 30 μL volume per animal. On days 3 and 5 after immunization (dpi.), the viral load in the lungs was assessed by the titration of lung suspensions in the MDCK cell culture with four mice per group.

For the immunogenicity and protection study, Balb/c mice were immunized with 6.0 log_10_ TCID_50_ by intranasal administration of 30 μL vector samples. The control mock-immunized group received an equivalent volume of DPBS. The FI-RSV prototype was used to model the RSV vaccine-associated enhanced respiratory disease: mice received a 100 μL intramuscular injection of 5 μg FI-RSV with 20 μg aluminum hydroxide adjuvant. Animal weight was monitored daily for 14 days after immunization (dpi). On 9 dpi, lungs from five mice in each group were collected for T-cell response analysis. On 21 dpi, blood serum was collected to assess the antibody response. Four weeks after immunization, mice were intranasally challenged with 5.0 log_10_ PFU/mouse of the RSV A2 strain. On day 4 after RSV infection (4 dpch.), seven mice per group were euthanized to estimate the viral load. Histological examination and T-cell immune response assessment were performed on day 6 after RSV infection (6 dpch.).

### 4.7. Enzyme-Linked Immunosorbent Assay (ELISA)

Serum samples were assessed for the presence of F-protein-specific IgG. Purified RSV (5 μg/mL) or recombinant F-protein RSV A2 (5 μg/mL) was used as the coating antigen. Sorption of antigens was carried out overnight at +4 °C in 96-well plates Maxisorp (Nunc). After incubation of the plates with blocking buffer (DPBS containing 5% skimmed milk), serial dilutions of sera were added to the coated plates and incubated for 1 h at room temperature. Goat HRP vs. mouse IgG (H+L) HRP (Abcam, Cambridge, UK) was used as the conjugate. The plates were stained with a TMB solution as a substrate, the reaction was stopped by adding 1 N H_2_SO_4_. Absorbance was measured at 450 nm on a multiplate reader with LVF CLARIOstar monochromators (BMG LABTECH, Ortenberg, Germany).

### 4.8. Hemagglutination Inhibition Assay

Sera were treated with a Receptor Destroying Enzyme (Denka Seiken, Tokyo, Japan) according to the manufacturer’s instructions. Serial twofold dilutions of sera were prepared in 96-well U-shaped-bottom plates. A total of 25 µL/well of the viral antigen (4 hemagglutination units/25 µL) was added and incubated for an hour. Then, 50 μL of 0.5% chicken red blood cells was added and incubated for an hour.

### 4.9. Histopathology

The collected lungs were fixed in a 10% neutral buffered formalin solution and dehydrated in increasing concentrations of isopropanol. After dehydration, the lungs were embedded in paraffin, sectioned, and stained with hematoxylin and eosin. A Carl Zeiss AxioSkop 2 plus light microscope (Carl Zeiss, Ortenberg, Germany) was used for histological examination. Microphotographs were taken with an AxioCam ERc5s digital camera with AxioVision Rel software. 4.8 (Carl Zeiss, Ortenberg, Germany). Lung tissue was assessed for the severity of inflammation and focal accumulation of infiltrating cells on a 5-point scale. The percentage of damaged tissue was evaluated visually based on the ratio of the damaged areas to the total section area.

### 4.10. Lymphocytes Isolation and Stimulation

Mice were sacrificed by cervical dislocation. The chest cavity was opened, and the right ventricle was perfused with 10 mL of ice-cold DPBS (Biolot, Russia). Lungs were minced into small pieces with scissors and then digested with 0.5 mg/mL of collagenase (Sigma, Saint Louis, MO, USA) and 40 U/mL of DNAse solution (Sigma, USA). Following tissue digestion, cells were filtered through a 70 μm cell strainer. Erythrocytes were lysed with RBC lysis buffer (BioLegend, San Diego, CA, USA) according to the manufacturer’s protocol. Cells were seeded at a density of 1 х 10^6^ cells per well into flat-bottom 96-well tissue culture plates (Nunc, Denmark) in RPMI 1640 (Gibco, USA) medium containing 10% of fetal bovine serum (FBS, Gibco, USA) and 1% of penicillin/streptomycin solution (Gibco, USA). Cells were incubated with 5 μg/mL of TYMLTNSELL peptide corresponding to the RSV F_249–258_-protein epitope, brefeldin A (Biolegend, USA), and anti-mouse CD28 (Biolegend, USA) at 37 °С and 5% CO_2_ for 6 h to stimulate cytokine production. The peptide was synthesized by Verta Ltd. (St. Petersburg, Russia). A total of 10 mg of dry peptide was dissolved in 1 mL of DMSO (PanReac Applichem, Darmstadt, Germany) and stored in small aliquots at −20 °C. The purity of the peptide was >90%, as determined by high-performance liquid chromatography. Additionally, cells were stimulated with 5 μg/mL of split-virion influenza vaccine preparation generated from the strain A/Guangdong-Maonan/SWL1536/2019 (H1N1)pdm09, anti-mouse CD28, and brefeldin A (BioLegend, USA) for 24 h.

### 4.11. Flow Cytometry

To estimate the percentage of CD4+ and CD8+ T lymphocytes producing cytokines after peptide stimulation, cells were stained with CD8-PE/Cy7, CD4-PerCP-Cy5.5, CD44-BV510, CD62L-APC/Cy7, IFNγ-FITC, TNFα-BV421, IL10-PE/Dazzle594, and IL2-PE antibodies (BioLegend, USA) using the BD Cytofix/Cytoperm™ Kit (BD Biosciences, USA) according to the manufacturer’s instructions. Zombie Red viability marker (BioLegend, USA) was used to identify dead cells. True Stain reagent, containing antibodies to CD16/CD32, was used to block non-specific antibody binding (BioLegend, USA). Data were collected on a CytoFlex flow cytometer (Beckman Coulter, Brea, CA, USA). The results were analyzed using the Kaluza Analysis v2 software (Beckman Coulter, USA). Background values obtained from the non-stimulated cells were subtracted from the corresponding values of the stimulated samples before the statistical analysis to estimate the increase in the cytokine production levels upon peptide stimulation.

### 4.12. Statistical Analyses

Data were analyzed using RStudio Desktop 1.0.153 (RStudio Inc., Boston, MA, USA). Groups were compared using appropriate tests (one-way or two-way ANOVA with Tukey’s post hoc test). A difference was considered statistically significant at *p* < 0.05.

## 5. Conclusions

In conclusion, our results highlight the importance of fine-tuning modifications while designing vaccine vectors to achieve the desired immune response to a specific pathogen. We have shown that the inclusion of the IgGκ signal peptide into the F-protein-derived transgene improves the protective efficacy of influenza NS vectors against RSV infection. Although we cannot affirm that the RSV antigen used in our study is optimal, the results obtained allow us to further optimize the structure of influenza NS vectors for expressing other transgenes. Notably, we found that the optimal vector NS-2AsF stimulated the highest level of CD8+ effector cells including a subset of lymphocytes co-producing IFNγ and IL10. In addition, immunization with this vector stimulated the accumulation of more Treg cells in the lungs upon RSV infection.

## Data Availability

The data presented in this study are available on reasonable request from the corresponding author.

## Supplementary Materials

The supporting information can be downloaded at: https://www.mdpi.com/article/10.3390/ijms241411445/s1.
