# Supplementary material for: IgGκ Signal Peptide Enhances the Efficacy of an Influenza Vector Vaccine against Respiratory Syncytial Virus Infection in Mice
Source: Int J Mol Sci. 2023 Jul 14;24(14):11445. doi: 10.3390/ijms241411445 (PMC10380829; doi:10.3390/ijms241411445)
Supplement: Supplementary file 1 [file ijms-24-11445-s001.zip › ijms-2480686-supplementary.pdf]

ijms-2480686-supplementary

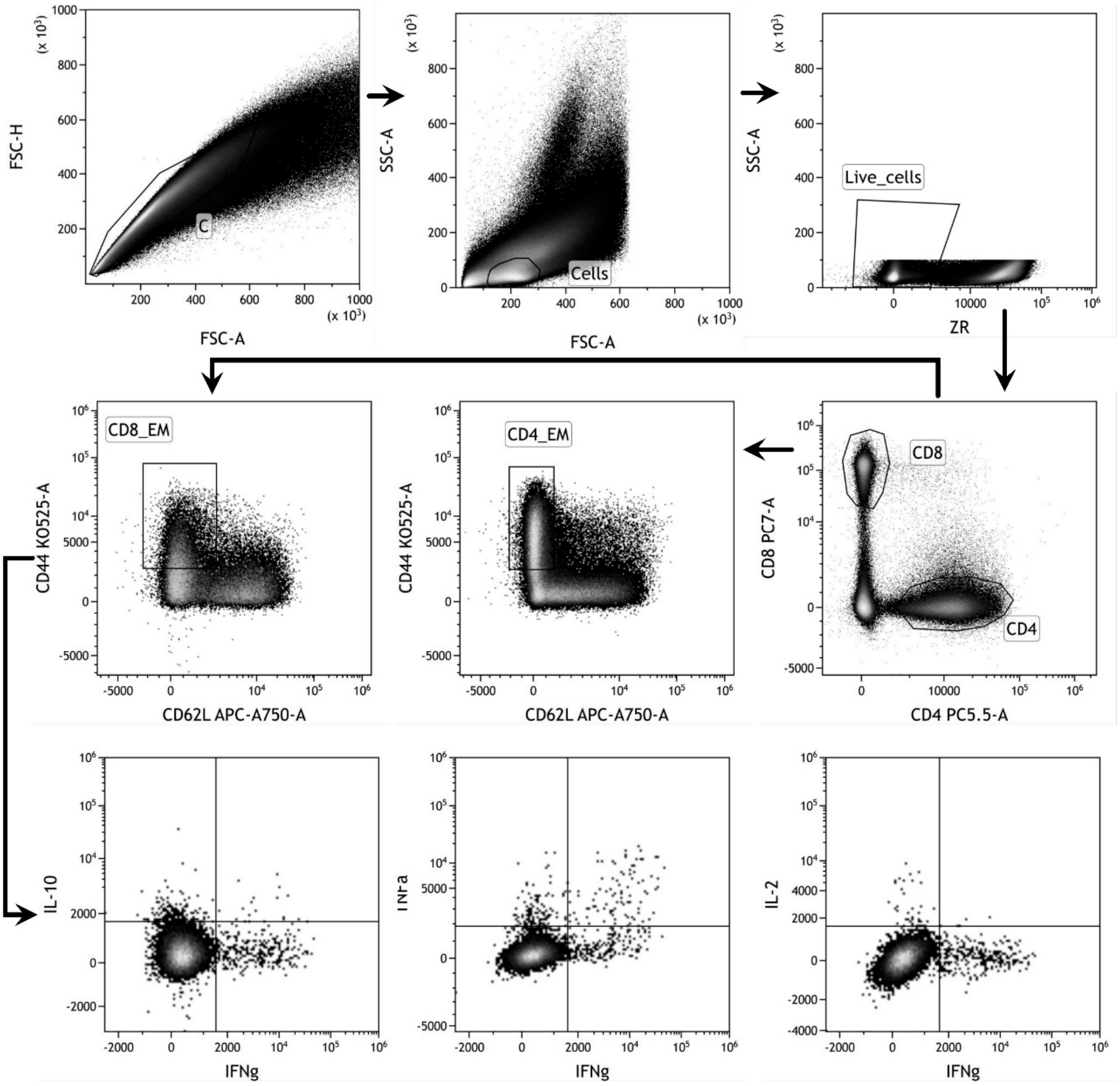

Figure S1. Gating scheme for flow cytometry analysis. After clipping of FSC-A/FSC-H doublets and isolation of a population of living single cells based on light scattering characteristics (FSC-A/SSC-A - cell gate). Based on Zombie Red fluorescence, non-viable cells are excluded from the analysis. The population of living cells was divided into two main subpopulations of T-lymphocytes: T-helpers (CD4+) and cytotoxic T-cells (CD8+). Based on the presence of CD44 and CD62L markers, subpopulations of effector (EM) memory T cells were isolated and characterized by the ability to produce cytokines (IFNγ, TNFα, IL2, IL10).

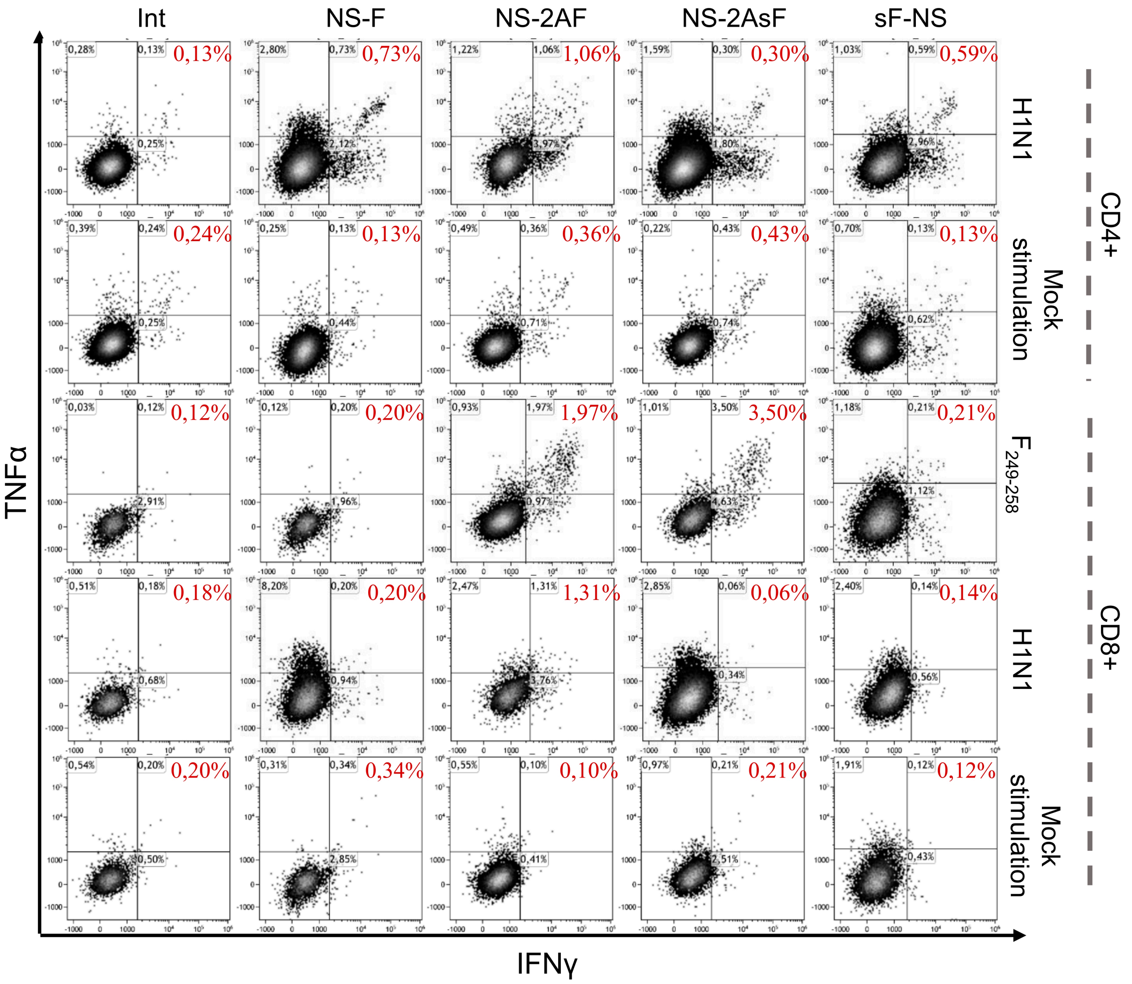

Figure S2. Typical plots showing the INF-γ and TNF-α profiles of CD4+ and CD8+ effector memory subsets lymphocytes in mouse lungs 9 days after intranasal immunization. Restimulation with influenza (H1N1)pdm09 strain were performed for 24 h or with the $F_{249-258}$ RSV-epitope for 6 h.

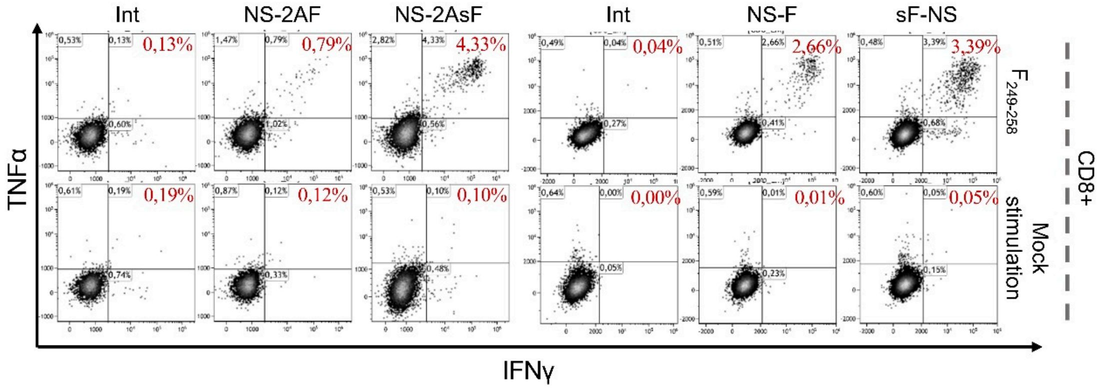

Figure S3. Typical plots showing the INF-γ and TNF-α profiles of CD8+ effector memory T lymphocytes in mouse lungs 21 days after intranasal immunization. Restimulation with the $F_{249-258}$ RSV-epitope were performed for 6 h.

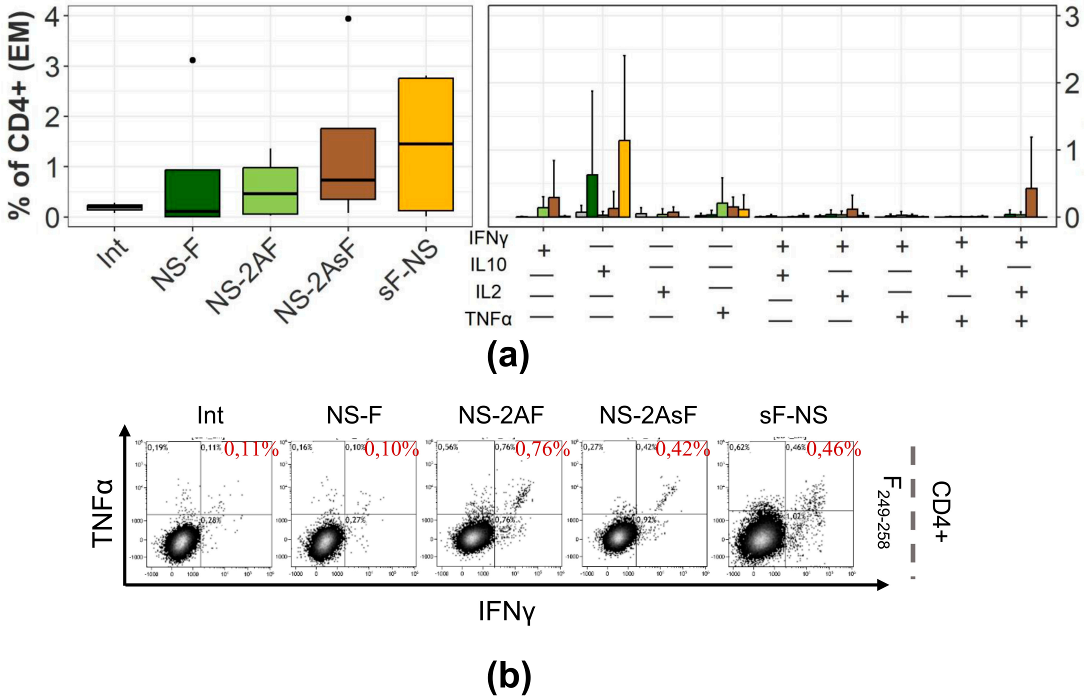

Figure S4. Adaptive T-cell immune response in mice lungs 9 days after intranasal immunization. **(a)** Boxplots on the left represent the the cumulative sum of all the effector memory CD4+ T cells producing at least one cytokine IFNγ/IL2/IL10 6 h after in vitro stimulation with the RSV $F_{249-258}$ epitope. Bar charts with error bars represent the percentage of different subpopulations of cytokine-producing T cells. **(b)** Typical plots showing the INF-γ and TNF-α profiles of CD4+ effector memory T lymphocytes in mouse lungs 9 days after intranasal immunization. Restimulation with the $F_{249-258}$ RSV-epitope were performed for 6 h.

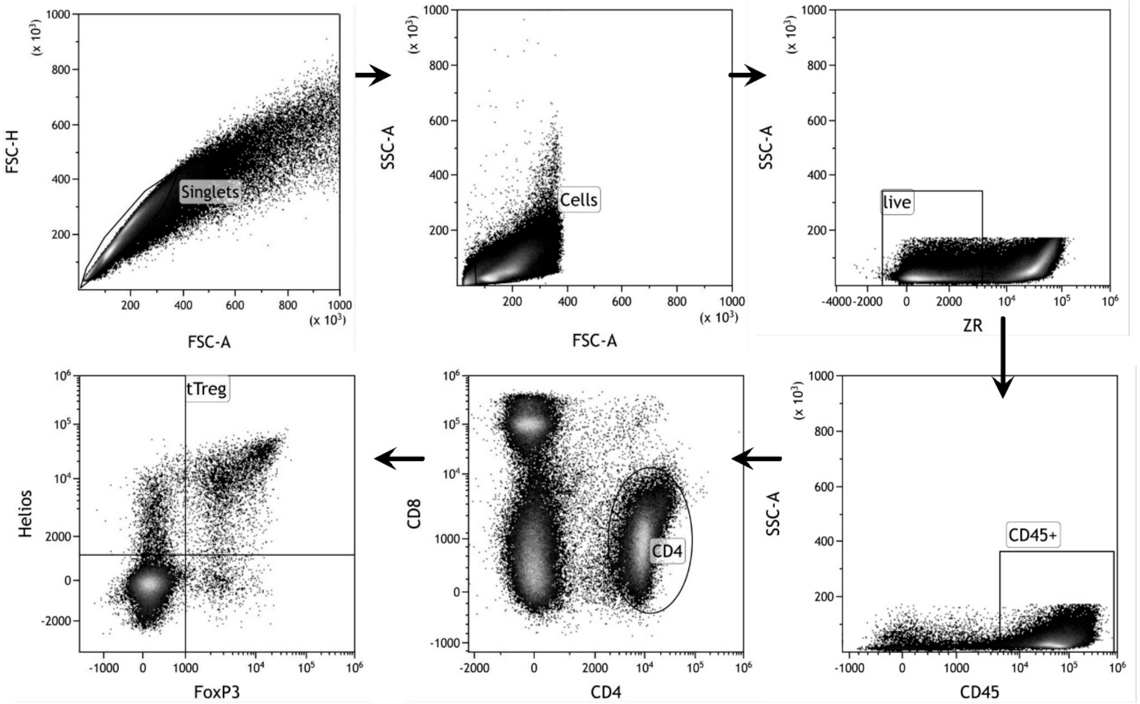

Figure S5 Gating strategy for the detection of regulatory T cells in the lungs of mice. The T-regulatory cells were defined in the subpopulation of

CD4+ T cells as a population simultaneously positive for FoxP3 and Helios (tTreg).

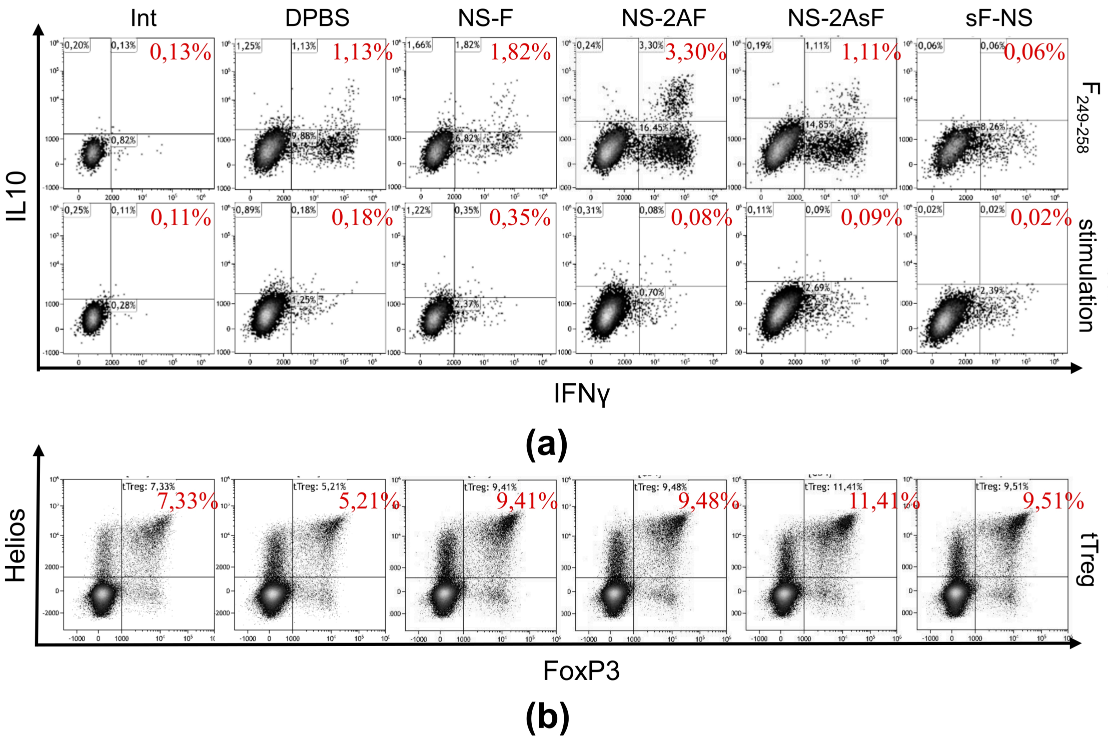

Figure S6. Typical plots **(a)** show the INF-γ and IL-10 profiles of CD8+ effector memory subsets after stimulation with RSV F249-258 epitope. **(b)** Typical plots present regulatory T cell.
